# Military Surgery at Santiago

**Published:** 1898-09

**Authors:** N. Senn

**Affiliations:** Chief of the Operating Staff in the Field


### Progress in Medical Science.

### MILITARY SURGERY AT SANTIAGO.

AS OBSERVED BY LIEUT. COL. N. SENN, M.D., U. S. V., CHIEF OF THE OPERATING STAFF IN THE FIELD.

LIEUT. COL. SENN, M. D., U. S. V., chief of operating staff with the army in the field, has published in the *Medical Record* a most interesting article on Recent experiences in military surgery after the battle of Santiago.

This report is particularly apropos coming as it does so close after the innumerable and confusing newspaper articles on the effects of the modern bullet, and it clears up much of the doubt which has existed regarding the action of the Mauser ball used by the Spaniards.

Dr. Senn found that the small jacketed bullet seldom carried into the tissues clothing or other infected substances. Most soft tissue wounds, uncomplicated by visceral lesions, healed by primary intention in a short time. When infection followed it was usually confined to the superficial portion of the wound; the wound of exit was more frequently affected than the wound of entrance. Wound infection was due to inadequate supply of first dressings; faulty application of first dressings; unnecessary change of dressings.

The evils of "meddlesome surgery" were apparent during the Cuban campaign. Dressings were changed too often and evil results followed frequently.

Bullets imbedded in soft tissues were usually found loose in small cavities filled with liquid blood or bloody serum and with evidences of encapsulation. The new bullet will become encapsulated more readily than the old leaden bullet.

In 10 per cent. of the wounded the bullet was found in the tissues. This was unexpected and was found to be due to the harum-scarum marksmanship of the followers of the flag of fire and blood. The condition of the recovered bullets showed beyond doubt that they were deflected or had passed through a resisting medium before striking the object for which they were intended.

Dissection and the *x*-ray have superseded the probe. The probe was never used on the Relief, the *x*-ray locating the ball. Dr. Senn found the course of the new bullet in the great majority of cases to be straight.

The wounds are classified in the report into, (1) the head; (2) the neck; (3) the spine; (4) the chest; (5) the abdomen; (6) the extremities.

In wounds of the head, death, when not instantaneous, was due to intracranial infection, encephalitis and leptomeningitis constituting the fatal complications. Intracranial inflammation was announced by cerebral hernia.

In spinal wounds death follows serious laceration of the cord. Death is due to septic leptomeningitis or sepsis and exhaustion from decubitus.

In chest wounds the chances of recovery are better now for the reason that the small ball does not carry with it foreign material. Except where the hemorrhage was severe the symptoms were mild. Treatment was by the expectant plan; in no case was the pleural cavity opened.

In abdominal wounds the campaign, he says, has "more than ever confirmed my convictions that not infrequently cases of penetrating gunshot wounds of the abdomen will recover without active surgical interference."

"For years," says Dr. Senn, "I have maintained, as the result of clinical experience and experiments on the cadaver, that a bullet may pass through the abdomen on a level with and above the umbilicus in an antero-posterior direction without producing visceral injury demanding operative intervention."

Four laparatomies were made at the fiist division hospital; all died. These were the only cases during the Cuban campaign to Dr. Senn's knowledge.

A numbei of perforating wounds of the abdomen were on a fair way to recovery without operation before they were sent home on transports.

In wounds of the extremities few primary amputations were made. A number of thigh and leg wounds, which became infected, are being treated by establishing free tubular drainage and resorting to antiseptic irrigation.

Dr. Senn reports a number of very interesting cases illustrative of the various classes of wounds and his latest work is a decided acquisition to surgical literature. His complete report will be valuable indeed to the student of modern surgery.

